# Editorial: Venoms and Toxins: At the Crossroads of Basic, Applied and Clinical Immunology

**DOI:** 10.3389/fimmu.2021.716508

**Published:** 2021-06-24

**Authors:** Manuela B. Pucca, Bryan G. Fry, Marco A. Sartim, Steve Peigneur, Wuelton M. Monteiro

**Affiliations:** ^1^ Medical School, Federal University of Roraima, Boa Vista, Brazil; ^2^ Venom Evolution Lab, School of Biological Sciences, University of Queensland, St Lucia, QLD, Australia; ^3^ Department of Research, Dr. Heitor Vieira Dourado Tropical Medicine Foundation, Manaus, Brazil; ^4^ Institute of Biological Sciences, Federal University of Amazonas, Manaus, Brazil; ^5^ Toxicology and Pharmacology, KU Leuven, Leuven, Belgium; ^6^ School of Health Sciences, Amazonas State University, Manaus, Brazil

**Keywords:** animal venoms, immunology, toxins, envenoming, drug development

Animal venoms are a rich source of biologically active molecules, and represent an important field in toxinology given its medical importance and bioprospecting potential of novel drugs. The studies explore a wide range of areas of knowledge, from animal biology to biomedical and chemical aspects of venom toxins and their biological effects including biochemistry, pathology, molecular biology, pharmacology, and others. In particular, immunology represents an essential field in toxinology, in which basic and clinical research covers four main pillars: pathophysiology, diagnosis, treatment, and drug discovery. In this Research Topic, authors from all over the world present new data or reviews regarding the effects triggered by toxins from a variety of venoms in the immune system, besides presenting new approaches for antivenom development and diagnosis.

## Pathophysiology

It is well established that immunological bases of envenomation pathophysiology are possibly one of the most studied topics in toxinology. Ryan et al. presented a review article on the major immunopathological aspects induced by animal venoms. The work consists of a detailed review of the latest efforts in the literature, covering innate and adaptative responses to envenomation as well as the toxin-induced effects on tissue cellular components and leukocyte populations. Among the topics, the authors approached mechanistic aspects of toxins recognition directly or indirectly (via tissue-damaged products) by the immune system and the pathological and protective responses towards venom compounds in order to neutralize the aggressor and symptom resolution.

The inflammatory response corresponds to a major issue considering its relevance to local and systemic damage caused by animal venoms, which the investigation of the mechanisms involved corresponds an essential aspect to better understand pathophysiological aspects of envenomation. Caterpillar envenoming is responsible for cutaneous reactions, and among its manifestations, the development of osteochondritis is associated with severe cases of envenoming. In order to better understand the inflammatory mechanism on joints, Villas-Boas et al. evaluated the *Premolis semirufa* (found in the Amazon forest region) hair extract on human chondrocytes culture. The authors demonstrated that venom extracts were capable of increasing the production of inflammatory mediators such as cytokines, chemokines, eicosanoids, and complement components, as well as matrix metalloproteinases and HMGB1, and reduced the expression of cartilage components aggrecan and type II collagen. Moreover, using transcriptome analysis, the study identified pathways associated with the inflammatory process of joint diseases, such as the inflammatory response, chemotaxis of immune cells, and extracellular matrix remodeling, evidencing an osteoarthritis-like signature.

Scorpion stings are responsible for the most cases of envenoming by venomous animals worldwide. Among the main pathological aspects, pain represents an important clinical manifestation in scorpion envenoming, which is associated with the direct action of neurotoxins on ion channels as well as by the inflammatory process induced by the venom. Abreu et al. carried out a pioneering study with the venom of *Rhopalurus crassicauda*, ​​an endemic species that inhabits exclusively the northernmost state of Brazil. The authors demonstrated that the venom and its isolated β-neurotoxin, Rc1, were capable to modulate the production of inflammatory cytokines and NFκ-B activation by macrophages, besides inducing a nociceptive behavior in mice. Rc1 was also shown to activate the voltage-dependent ion channels Nav1.4, Nav1.6, and BgNav1. The authors also demonstrated that scorpion antivenom used in Brazilian health units were unable to recognize *Rhopalurus crassicauda* venom and Rc1, indicating the need for different approaches for the treatment of envenomings regarding *Rhopalurus crassicauda* accidents.

Complement activation induced by snake venoms has been widely reported. Silva-de-França et al. investigated the effects of *Naja annulifera* venom on the complement system and explored its contribution to envenomation pathological aspects. Using *in vitro* and *in vivo* assays, the authors found that the venom was capable to induce an inflammatory response, characterized by local and systemic reactions (leukocyte influx, oedema, acute lung injury, and production of inflammatory mediators) in a complement-dependent manner. The research group also indicated the C5a-C5aR1 axis as a possible therapeutical target for *Naja annulifera* snakebite treatment.

The evaluation of circulatory inflammatory mediators in patients has brought important information on the inflammatory behavior of envenomation, guiding the search for novel biomarkers. Gerardo et al. introduce a novel prognostic model by associating inflammatory mediators (obtained pre and post antivenom therapy) and clinical features to functional recovery up to 28 days of patients from Crotalinae snakebite in the United States. The authors discovered biomarkers networks, as well as clinical variables, to predict patient recovery and increase patient prognosis as a strategy to be applied in the clinical management of snakebite. During animal envenomation, much is discussed around the direct effects of inflammation and its association to local tissue damage. However, the inflammatory response is also responsible for interfering in other physiological systems, such as hemostasis known as the inflammation-coagulation crosstalk. The study performed by Wellmann et al. investigated the correlation of circulating inflammatory cytokines and chemokines to fibrinogen levels observed in victims of the lancehead *Bothrops atrox* snakebite in North Brazil. The authors have found distinguished cytokines/chemokines profile between patients presenting normal and decreased levels of fibrinogen, highlighting that patients with hypofibrinogenemia showed increased levels of CCL-5 and decreased INF-γ, as well as a negative correlation between CXCL-8 and IL-2 to fibrinogen levels. These data support the relationship of the immune response and hemostatic disturbances observed in the envenomation pathophysiology, and the use of inflammatory mediators as possible biomarkers of coagulopathy prognosis.

## Diagnosis

Despite the great advance in the studies of the pathophysiological aspects of snakebite, and the established protocols for clinical management and treatment, an important issue that is neglected is the snakebite diagnosis. The snakebite diagnosis is based majorly on the patient’s clinical aspects and the accident history to identify the aggressor. Knudsen et al. performed a review article exploring clinical and epidemiological aspects of snakebite worldwide and focused on research efforts within snakebite diagnosis. The study brings important data on different technological strategies for the diagnosis of snakebite and discuss new trends in the development of novel and rapid methods for snakebite diagnosis in order to promote a faster recovery and shorter time to hospital discharge.

## Treatment

The unique specific treatment available for treating envenomings caused by venomous animals is the use of heterologous antivenoms. These antivenoms are composed of preparations containing IgGs or derived-fragments from the plasma of hyperimmunized animals. Although antivenoms have been produced using a century technique, conventional antivenoms are currently being used to save countless lives worldwide.

Most commercial antivenoms are polyspecific, meaning that they are obtained from animals immunized with a mixture of venoms from more than one species. However, based on similarities of venom toxins, antivenoms can cross-neutralize toxins from species that were not used in the immunization protocol (paraspecific neutralization). Thus, although there are known only regional and national antivenom preparations (depending on the composition of the venomous animal fauna), the development of universal antivenoms would be of immediate benefit. Ratanabanangkoon reviewed the possibility of manufacturing a plasma-derived antivenom (PDAV) against neurotoxic snake venoms. The review discusses that with the correct diverse toxin repertoire composed of a careful selection of elapid toxins, a universal PDAV against elapid neurotoxic venoms can be produced and used for the treatment of envenomation by elapid snakes at a global level.

On the other hand, it is well-explored that heterologous antivenoms can trigger both acute (anaphylactic or pyrogenic) and delayed (serum sickness) reactions, evidencing the urgent need for improving the specific treatment for envenomings caused by venomous animals. Fortunately, with the advent of novel antibody discovery technologies and improved methods for protein engineering, there are now several advances in the field. Føns et al. reported the first recombinant human monoclonal immunoglobulin G antibody that binds α-latrotoxin (from the Mediterranean black widow spider *Latrodectus tredecimguttatus*) and shows its *ex vivo* neutralization efficacy.

Independent of the type and nature (heterologous or homologous) of the antivenoms, their efficacy is evaluated at the preclinical level by assessing its capacity to neutralize the lethal action of venoms in animal models (e.g. mice). Therefore, there is a growing awareness of the need to significantly reduce the number of mice used based on the 3Rs (Replacement, Reduction and Refinement) philosophy. Gutiérrez et al. performed a systematic review about the development of *in vitro* tests for antivenom preclinical efficacy assessment, focusing mainly on studies in which the correlation between *in vitro* and *in vivo* tests was evaluated.

The variability in the venom composition among snakes from the same genus or species is directly associated with possible limitations on antivenoms effectiveness. Seneci et al. have demonstrated that geographical and ontogenic variations in venom composition of Mexican rattlesnakes implicate different coagulotoxicity. The authors demonstrated that Antivipmyn^®^, the major antivenom used in Mexico for rattlesnake envenoming, presented drawbacks in neutralizing the true procoagulant activity (mediated by factor X activation), differently from prinomastat (a matrix metalloprotease inhibitor), which was capable of inhibiting stable clot formation induced by factor X activating metalloproteases.

Pucca et al. have shown that in South America, there are some differences in relation to the venom composition and clinical pictures resulting from different subspecies of *C. durissus.* For instance, envenoming caused by *C. d. ruruima*, an important public health issue in the northernmost state of Brazil, Roraima, evolve with a higher frequency of unclottable blood and bleeding compared to other *C. durissus* subspecies, thus deserving special attention in the therapeutic management using the available antivenoms. Authors discuss the efficacy of the Brazilian horse-derived antivenoms to treat *C. d. ruruima* envenomings, focusing on the discovery and development of monoclonal antibodies against *Crotalus* toxins to design recombinant antivenoms based on oligoclonal mixtures of antibodies targeting all medically important venom toxins. Likewise, Chowdhury et al. have demonstrated that clinically significant variations in snakebite effects and antivenom neutralization efficacy are underpinned by the dynamic evolution of the Palearctic vipers’ venoms (Daboia, Montivipera, and Vipera genera). Testing of antivenom neutralization by three commercial antivenoms (Inoserp Europe, VIPERFAV, and ViperaTAb) revealed differential intra-species and inter-species ability to neutralize the lethal FX activating effects, reflective of variations in the composition of the venoms used in the immunizing processes.

Several efforts have been performed in order to improve the treatment of snakebite, such as the search for novel drugs or antivenoms improvement in order to neutralize venom toxins. However, the strategy for the treatment of clinical manifestations has gained a spotlight with the advent of biological techniques. Sanchez-Castro et al. performed a review article on the use of mesenchymal stromal cells (MSCs) as a promising treatment for muscle regeneration following a snakebite, detailing the local damage pathophysiology during snakebite and the benefits of MSCs due to its anti-inflammatory and pro-regenerative properties. Moreover, the authors also presented an experimental section with original data on the applicability of a MSCs protocol developed by the research group on *Bothrops atrox* experimental envenoming in mice, showing a potential beneficial effect on muscle damage.

## Drug Discovery

Aside from the role of venom components on envenomation pathogenesis, toxins are important candidates in the prospection of novel biomolecules with biotechnological and therapeutical relevance. Among them, crotoxin, the major toxic component of the venom of South American rattlesnake *Crotalus durissus*, has been in the spotlight due to its immunotherapeutic properties concerning its anti-inflammatory and immunosuppressive behavior. Sant’anna et al. conducted a study on the potential of a nanoformulation complexed with crotoxin to treat multiple sclerosis in an experimental autoimmune encephalomyelitis model. The nanoformulation was more efficient in reducing pain, ameliorate motor impairment and mitigate peripheral Th17 response and cell infiltration to the central nervous system, compared to animals treated with crotoxin alone.

Another applicability of venom toxins in medicine comprises the use of toxins as coadjuvant molecules, without a direct pharmacological-target purpose to treat the patients. Abbade et al. conducted a phase I/II non-randomized, single-arm clinical trial using heterologous fibrin sealant for the treatment of chronic venous ulcers. Fibrin sealants are widely used in dermatological treatments, due to their tissue repair properties, which the authors used a formulation containing heterologous fibrinogen-rich cryoprecipitate and a thrombin-like toxin from the venom o *Crotalus durissus* as the inducer of the fibrin formation. The study showed promising results, which the bioproduct proved to be safe and non-immunogenic with good preliminary efficacy for the treatment of chronic venous ulcers.

In summary, studies of venom components reveal more and more examples of natural toxins with actions on the immune system, which can be used both as pharmacological tools and as a useful starting point for drug development. Moreover, understanding the effects triggered by venoms and their toxins is particularly important to elucidate the mechanism of clinical envenomings and help in the discovery of better antivenoms and ancillary treatments.

## Author Contributions

All authors listed have made a substantial, direct and intellectual contribution to the work, and approved it for publication.

## Funding

We thank Conselho Nacional de Desenvolvimento Cientófico e Tecnológico (CNPq, The National Council for Scientific and Technological Development, scholarship to MBP no. 307184/2020-0 and WMM n. 309207/2020-7). WMM acknowledges funding support from Fundação de Amparo à Pesquisa do Estado do Amazonas (PAPAC 005/2019, PRÓ-ESTADO, and Posgrad calls). MBP acknowledges funding support from Hamish Ogston Foundation - Global Snakebite Initiative.

## Conflict of Interest

The authors declare that the research was conducted in the absence of any commercial or financial relationships that could be construed as a potential conflict of interest.

